# A two-pore channel protein required for regulating mTORC1 activity on starvation

**DOI:** 10.1186/s12915-019-0735-4

**Published:** 2020-01-22

**Authors:** Fu-Sheng Chang, Yuntao Wang, Phillip Dmitriev, Julian Gross, Antony Galione, Catherine Pears

**Affiliations:** 1 Department of Biochemistry, University of Oxford, South Parks Road, Oxford, OX1 3QU UK; 2 Department of Pharmacology, University of Oxford, Mansfield Road, Oxford, OX1 3QT UK

**Keywords:** Two-pore channel (TPC), mTORC1, *Dictyostelium*, Acidic vesicles, Autophagy

## Abstract

**Background:**

Two-pore channels (TPCs) release Ca^2+^ from acidic intracellular stores and are implicated in a number of diseases, but their role in development is unclear. The social amoeba *Dictyostelium discoideum* proliferates as single cells that aggregate to form a multicellular organism on starvation. Starvation is sensed by the mTORC1 complex which, like TPC proteins, is found on acidic vesicles. Here, we address the role of TPCs in development and under starvation.

**Results:**

We report that disruption of the gene encoding the single *Dictyostelium* TPC protein, TPC2, leads to a delay in early development and prolonged growth in culture with delayed expression of early developmental genes, although a rapid starvation-induced increase in autophagy is still apparent. Ca^2+^ signals induced by extracellular cAMP are delayed in developing *tpc2*^*−*^ cells, and aggregation shows increased sensitivity to weak bases, consistent with reduced acidity of the vesicles. In mammalian cells, the mTORC1 protein kinase has been proposed to suppress TPC channel opening. Here, we show a reciprocal effect as *tpc2*^*−*^ cells show an increased level of phosphorylation of an mTORC1 substrate, 4E-BP1. mTORC1 inhibition reverses the prolonged growth and increases the efficiency of aggregation of *tpc2*^*−*^ cells.

**Conclusion:**

TPC2 is required for efficient growth development transition in *Dictyostelium* and acts through modulation of mTORC1 activity revealing a novel mode of regulation.

## Background

Calcium (Ca^2+^) is a ubiquitous signalling ion in eukaryotic cells [1]. Cytosolic Ca^2+^ levels are kept low by pumps that remove cytosolic Ca^2+^ from the cell or into intracellular compartments. Transient release into the cytosol via gated channels is vital to control a variety of important cellular processes including development and differentiation. Intracellular Ca^2+^ stores are defined as neutral (gated by IP_3_ and ryanodine receptors) or acidic (gated by TRPML and TPC channels), and release from the stores is differentially regulated. Two-pore channel proteins (TPCs) are members of the voltage-gated ion channel superfamily [2, 3]. There are up to three isoforms of TPCs found on acidic compartments, comprised mainly of lysosomes and the endosomal system [4, 5]. They are cation channels mediating NAADP-dependent Ca^2+^ release from acidic organelles [6], also regulated by the inositol lipid PI(3,5)P_2_ and by phosphorylation [7]. TPC1 is also voltage gated. TPCs have been implicated in a number of disease states including diabetes and cancer and are required for Ebola infection [8]. They also play an important role in development and differentiation in a number of systems, including zebrafish where the loss of TPC2 attenuates differentiation of the skeletal and smooth muscles [9].

Mammalian multicellular development cannot be easily replicated in tissue culture and is currently most frequently studied in mice and, in terms of studying the role of intracellular Ca^2+^, complicated by the fact that mice express multiple variants of known channels. The social amoeba *Dictyostelium discoideum* circumvents these problems. This organism proliferates as single multipotent stem cells that, on starvation, form a multicellular organism and differentiate into two major cell types [10]. The final fruiting body contains a head of spores held aloft by a stalk comprised of highly vacuolated stalk cells. *Dictyostelium* expresses a simplified complement of Ca^2+^ channels compared to mammalian cells (for example, only one gene encoding a TPC protein compared to three in some higher eukaryotes) and a rapid developmental cycle (24 h) to facilitate analysis. In early development formation of the multicellular structure or aggregate from a field of amoebae is mediated by low (nM) pulses of extracellular cAMP which work through membrane receptors to induce changes in gene expression to drive the developmental programme. Signalling pathways activated downstream of extracellular cAMP include an increase in cytosolic Ca^2+^ [11]. This Ca^2+^ transient is severely compromised in the absence of IplA, a protein highly related to the IP_3_ receptor in mammalian cells, consistent with the majority of this Ca^2+^ being derived from the endoplasmic reticulum [12]. However, this does not rule out the existence of low Ca^2+^ transients below the current detection limits, which could be derived from acidic compartments. Indeed it has been proposed that low-level release from the acidic stores primes the channels on neutral stores for more wide-scale Ca^2+^ release [13].

As well as neutral stores (endoplasmic reticulum), *Dictyostelium* amoebae contain acidic Ca^2+^ stores (contractile vacuole system) (reviewed in [14]). Acidic compartments play a role in early *Dictyostelium* development as weak bases inhibit aggregation by giving rise to a marked increase in pH of acidic compartments whilst having little discernible effect on cytosolic pH [15]. Consistent with this, mutant strains with defects in V-type H^+^-ATPases have a developmental defect reminiscent of treatment with weak bases [16]. A number of Ca^2+^ channels associated with acidic stores are found in *Dictyostelium* including TPC2, TRPML and P2X receptors. The P2XA receptor is responsible for osmotic regulation [17, 18], and cells lacking TRPML (mucolipin) protein show increased lysosome exocytosis [19]. However, no role for these channels in regulating development has been described.

Here, we report that disruption of the gene encoding the single *Dictyostelium* TPC protein, TPC2, leads to a delay in early development. Ca^2+^ fluxes induced by extracellular cAMP are maintained but slightly delayed in *tpc2*^*−*^ cells, but aggregation shows increased sensitivity to inhibition by weak bases, consistent with altered acidity of vesicles in *tpc2*^*−*^ cells. Loss of TPC2 alters gene expression prior to the onset of development and leads to an increased cell density in culture. In mammalian cells, the mTORC1 protein kinase is regulated by nutrient status, associated with acidic compartments and modulates TPC channel opening [20, 21]. Here, we show a reciprocal effect in that *tpc2*^*−*^ cells show an increased level of phosphorylated form of the mTORC1 substrate, 4E-BP1, both during the prolonged growth in shaking culture and following the onset of starvation. *Tpc2*^*−*^ cells show increased sensitivity to mTORC1 inhibitors during growth, and inhibitor treatment increases their efficiency of aggregation, consistent with the increased mTORC1 activity playing a role in the developmental phenotype of *tpc2*^*−*^ cells. Therefore, at the growth development transition in response to starvation in *Dictyostelium*, mTORC1 activity is maintained at a higher level in the absence of TPC2.

## Results

**Fig. 1:**
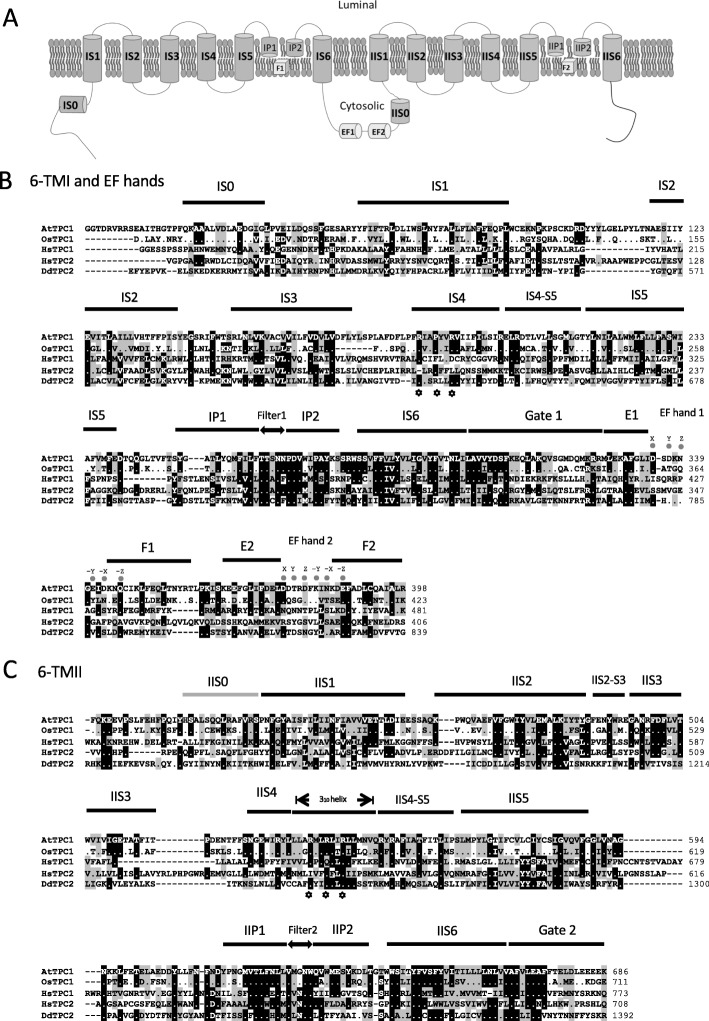
Domain sequence alignment of predicted TPC proteins. **a** Schematic illustration of TPC proteins, illustrating the 12 transmembrane domains (IS1–6 and IIS1–6), the pore regions (P1 and 2), the filter regions (F1 and 2) and the EF hands (EF1 and 2) [22]. TPC2 protein sequences from *Dictyostelium discoideum* (DdTPC2) aligned with *Arabidopsis* TPC1 (AtTPC1), rice TPC1 (OsTPC1) and human TPCs (HsTPC1 and HsTPC2) are shown. **b** The first six transmembrane domains and EF hands and **c** the second six transmembrane domains. The prediction of secondary structure is based on the published structure of AtTPC1 [22] [23]; except IIS0 which is predicted based on [24]. Stars illustrate potential voltage-sensing residues [23]. The sequences were aligned using ClustalW built-in BioEdit

TPC proteins in plants and animals have a distinctive domain organization (Fig. 1a) with two pore regions each consisting of six transmembrane domains with a pore region between the fifth and the sixth [22–24]. *Dictyostelium* contains a single gene predicted to encode a TPC protein (DDB_G0289105 dictybase.org), showing homology with human TPC1 and TPC2 (identity 25% and 29%, respectively), *Arabidopsis thaliana* TPC1 (27%) and *Oryza sativa* TPC1 (29%) (Fig. 1b, c). This higher degree of identity to TPC2 proteins has led to the *Dictyostelium* version being named TPC2 [25]. The signature two groups of the six transmembrane domains are well conserved, with a linker containing two putative EF hand domains predicted to bind Ca^2+^. Similar EF hands are found in both plant and mammalian TPC1s, although the mammalian ones lack conserved residues required for Ca^2+^ binding [24]. Alignment of the predicted ion conduction pore sequences shows that for the ion conductivity filter 1 in the pore 1 region, plant and animal TPC2s contain the consensus sequence TT[SA]N[NF] whereas *Dictyostelium* TPC2 contains TTCNF. In filter 2 in pore 2, *Dictyostelium* TPC2 contains the consensus sequence VLNNW similar to the VVNNW found in animal TPCs, in contrast to the VMGNW motif found in plant proteins. Plant TPC1s have been reported to be non-selective ion channels that conduct K^+^, Ca^2+^, Na^+^ and ions, whereas human TPC1 and TPC2 conduct both Na^+^ and Ca^2+^, and human TPC1 may also conduct H^+^ ions [6, 26, 27]. The sequence differences in these regions could determine the ion selectivity of the ion channel.

### Role for TPC2 in early development

**Fig. 2:**
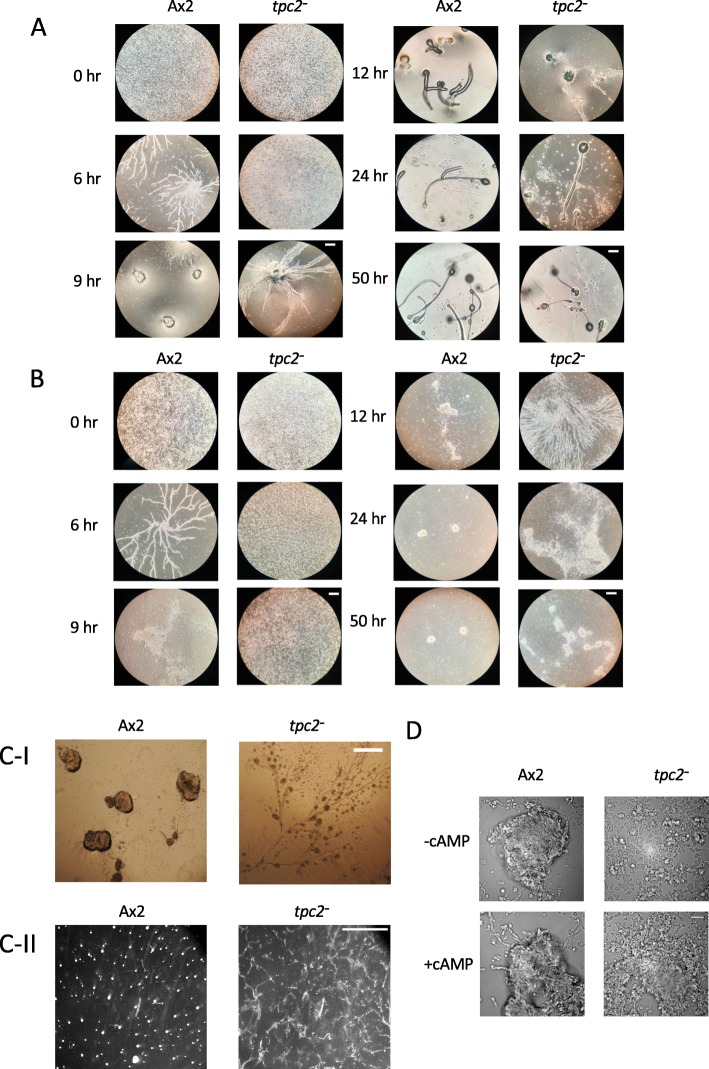
Developmental delay of *tpc2*^*−*^ cells. Parental Ax2 and *tpc2*^*−*^ cells were harvested from exponential growth and washed twice with HKC-LoCa buffer. **a** Agarose development. 3.84 × 10^6^ cells plated on 1% agarose HKC-LoCa buffer in a 6-well plate (final cell density 4 × 10^5^ cells/cm^2^), incubated at 22 °C. **b** Under buffer development, 3.8 × 10^6^ cells plated on 1% agarose HKC-LoCa buffer in a 6-well plate (final cell density 4 × 10^5^ cells/cm^2^), incubated at 22 °C. Developmental structures were observed at the times shown and photographed using a Coolpix P310 digital camera mounted on a light microscope (Motic AE21 Inverted Phase Microscope). Scale bars, 200 μm. Cells were developed as A (**c-I**) after 9 h or B (**c-II**) after 20 h, and representative images at low magnification are shown. Scale bars: **c-I** 200 μm, **c-II** 1 cm. Following development under buffer for 20 h (**c-II**) *tpc2*^*−*^ cells form 96 ± 4 aggregates/cm^2^ compared to 20 ± 2 aggregates/cm^2^ for Ax2 cells (average ± SEM of three independent experiments). **d** Cells were resuspended at 1.25 × 10^7^ cells/ml HKC-LoCa buffer and shaken for 2 h then pulsed with or without cAMP (50 nM every 6 mins) for 4 h, all at 22 °C. Cells were allowed to settle for 15 min then observed using DeltaVision Elite microscope and photographed using a EMCCD camera mounted on the microscope. Scale bar, 50 μm

To determine if TPC2 plays a role in development, *Dictyostelium* strains were generated in which the *tpc2* gene has been disrupted by homologous recombination removing the majority of the coding sequences, including that coding for the transmembrane domains (Additional file 1: Figure S1). Upon starvation on buffered agar *tpc2*^−^ strains showed a delay in early development (Fig. 2a) with long streams apparent suggesting large aggregation territories. However, once aggregates formed, development continued to generate apparently normal fruiting bodies. This delay was exacerbated when cells were developed under buffer, where aggregation was prolonged (Fig. 2b) with large aggregation territories and long streams apparent in *tpc2*^*−*^ strains. Even after 50 h of starvation, many cells failed to enter aggregates. In both developmental conditions, the long streams formed by *tpc2*^*−*^ cells broke up into small aggregates that then coalesced into fewer larger aggregates as development proceeded (Fig. 2c-I, c-II). Early development is dependent on the ability to generate and respond to pulses of extracellular cAMP. Exogenous pulses of cAMP did increase the efficiency of aggregate formation in *tpc2*^*−*^ cells but did not completely rescue aggregation, suggesting the cells may be deficient in both generating and sensing the cAMP signal (Fig. 2D).

### Ca^2+^ signalling in *tpc2*^*−*^ cells

As extracellular cAMP is known to generate a cytosolic Ca^2+^ response, the FRET-based Ca^2+^ sensor YC-nano15 [28] was expressed in parental Ax2 and *tpc2*^*−*^ cells to monitor cytosolic Ca^2+^. The YC-nano15 protein contains both YFP and CFP, and the distance between these is reduced by binding Ca^2+^ in the linker region. YC-nano15 senses very low Ca^2+^ levels with a *K*_*d*_ of 15 nM and, in agreement with previous reports [28], it was able to detect changes in cytosolic Ca^2+^ in *Dictyostelium* cells (Additional file 2: Figure S2). The average cytosolic Ca^2+^ level in growing cells was reduced in *tpc2*^*−*^ cells (average YFP/CFP ratio = 1.62 ± 0.29; *n* = 157) compared to parental Ax2 cells (average ratio = 2.04 ± 0.41; *n* = 181) (Additional file 3: Figure S3).

**Fig. 3:**
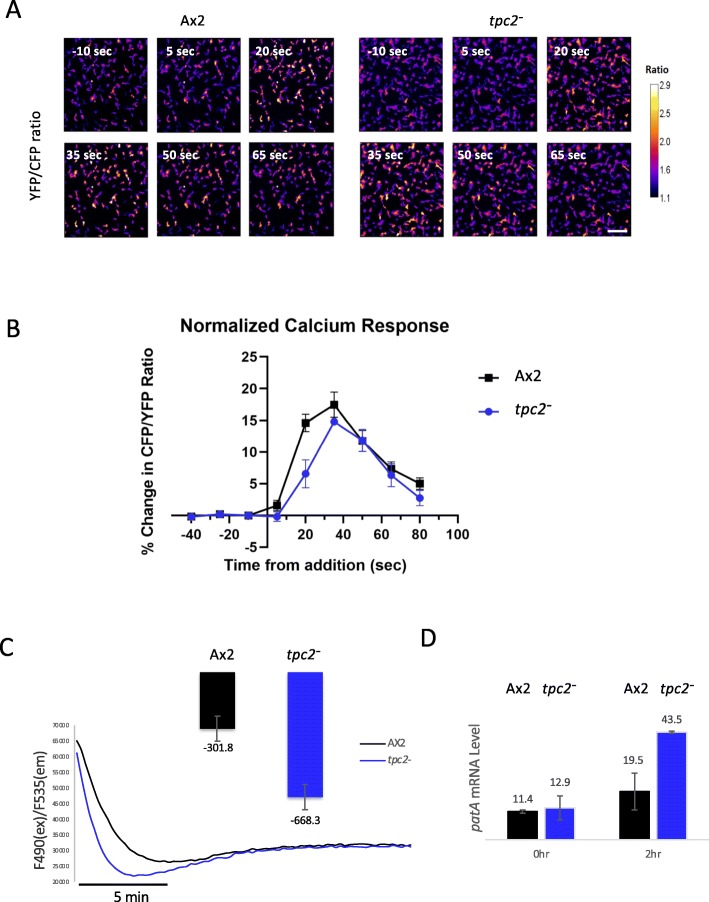
Ca^2+^ responses in *tpc2*^*−*^cells. **a** Ax2- and *tpc2*-null cells were developed at a density of 4 × 10^5^ cells/cm^2^ in HKC-LoCa buffer for 6 h (T6). The cells were stimulated with 50 nM cAMP at time 0. The [Ca^2+^]c level was recorded as YFP/CFP ratio images using a widefield fluorescence inverted microscope. The time (seconds) relative to cAMP addition is shown. Scale bar, 50 μm. One experiment representative of three. **b** In three individual experiments as described in A, the mean ratio was calculated at the times shown and the change in ratio compared to the cells 40 s prior to cAMP addition plotted (mean + SEM). **c** Vesicles were isolated from Ax2 and *tpc2*^−^ cells developed in shaking suspension in KK2 and EGTA for 4 h, with pulses of cAMP (50 nM) added every 6 min; 200 μg of the vesicles was added to 100 μl Ca^2+^ uptake buffer supplemented with 100 μM NaN_3_, 1.5 mM ATP and 6 μg/ml oligomycin A. Representative trace showing calcium uptake by vesicles made from Ax2 and *tpc2*^−^ cells and the average gradient of uptake from the three independent experiments. **d** mRNA was extracted from Ax2 or *tpc2*^−^ cells growing or developed in shaking suspension for 2 h and levels of *patA* expression quantified by qRT-PCR relative to expression of IG7. Results are the average of two independent experiments (mean + SEM)

To determine if the delay in aggregation seen in *tpc2*^*−*^ cells is due to a deficiency in cAMP-induced Ca^2+^ release, parental Ax2 and *tpc2*^*−*^ cells expressing YC-nano15 were developed for 4 h. The addition of 50 nM cAMP to parental Ax2 cells led to an increase in cytosolic Ca^2+^ levels (Fig. 3a, b) reaching near peak values at 20 s following cAMP addition. A comparable increase was also apparent in *tpc2*^*−*^ cells although the increase was delayed compared to Ax2 cells. Therefore, TPC2 channels are not required for the extracellular cAMP-induced increase in cytosolic Ca^2+^, but their loss alters the kinetics of the response.

### Ca^2+^ uptake in vesicles derived from *tpc2*^*−*^ cells

A mixed population of vesicles containing both neutral and acidic stores were generated from Ax2 and *tpc2*^*−*^ cells developed with exogenous cAMP pulses for 4 h in the presence of EGTA to deplete the stores of intracellular Ca^2+^. The vesicles were then incubated in a Ca^2+^ containing buffer in the presence of a Ca^2+^ indicator (Fluo-3) and the rate of decrease in fluorescence monitored as the Ca^2+^ entered the vesicles to replenish the stores. The rate of Ca^2+^ uptake was consistently higher in vesicles prepared from *tpc2*^*−*^ cells than from parental Ax2 cells (Fig. 3c). This suggests that either TPC2 channels normally act as Ca^2+^ leak channels which are absent in the vesicles from *tpc2*^*−*^ cells or loss of TPC2 leads to an alteration in the level or activity of Ca^2+^ pumps found in these vesicles, or both. The pump responsible for maintaining the ion balance of acidic vesicles in *Dictyostelium* is Pat1, a P-type Ca^2+^ transporting ATPase [29]. Quantification of mRNA levels revealed an increase in *patA* mRNA encoding Pat1 in *tpc2*^*−*^ cells in early development (Fig. 3d), which, if this leads to increased protein levels, is one potential mechanism to explain increased Ca^2+^ uptake.

Vesicles prepared from developing *Dictyostelium* cells release Ca^2+^ in response to arachidonic acid [30], although the mechanism for this release has not been defined. Vesicles derived from *tpc2*^*−*^ cells show comparable sensitivity to arachidonic acid-induced Ca^2+^ release as vesicles from Ax2 cells, so TPC2 is not directly responsible for this release (Additional file 4: Figure S4).

### Reduced acidity of vesicles in *tpc2*^*−*^ cells

**Fig. 4:**
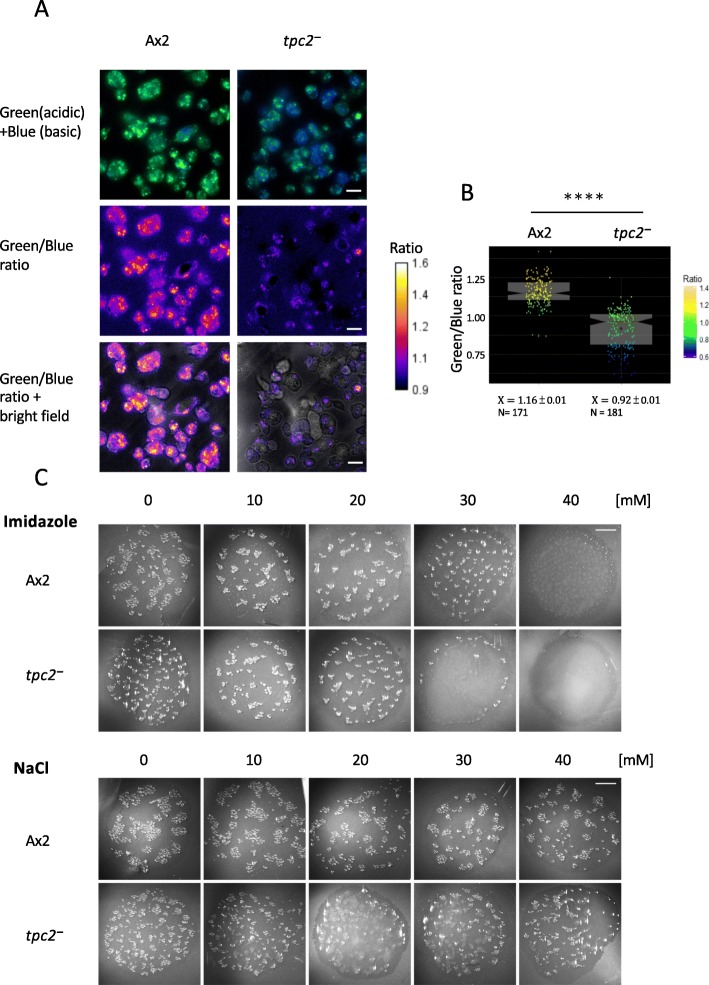
Altered properties of acidic vesicles in *tpc2*^−^ cells. **a** Intracellular acidic organelles were visualized using a fluorescent LysoSensor™ Yellow/Blue DND-160 probe. Cells were harvested from exponential growth, washed twice with HKC-LoCa buffer and plated onto a 35-mm ibidi μ-Dish at a final cell density at 4 × 10^5^ cells/cm^2^. The cells were allowed to develop for 4 h. The PDMPO probe was added at a final concentration of 2 μM for 15 mins. LysoSensor™ emits predominantly yellow fluorescence, and in less acidic organelles, it emits blue fluorescence [32]. Ratio images are shown and merged with bright field images. Cells were observed using DeltaVision Elite microscope and photographed using an EMCCD camera. Scale bars, 10 μm. **b** Quantification and statistical analysis (Student *t* test) of ratios. $\overline{X\ }$, sample mean; *N*, sample numbers; ****P ≤ 0.0001. **c** Ax2 and *tpc2*^−^ cells were harvested from exponential growth, and 5 × 10^7^ cells were plated on filters incubated on paper presoaked LPS pH 7.0 in the presence of various concentrations of the weak bases imidazole or sodium chloride at 22 °C. Aggregates were observed using a Leica MZ FL III Stereomicroscope and photographed using a Hamamatsu digital camera ORCA-05. Scale bars, 5 mm. One experiment representative of three

The altered uptake of Ca^2+^ in vitro and the increased expression of *patA* mRNA raised the possibility that, rather than a direct effect on cytosolic Ca^2+^ changes, the effect of TPC2 on early development was mediated by an alteration in acidic vesicle properties. In *Dictyostelium*, these vesicles are highly acidic [31]. Despite this, a decrease in the acidity of the vesicle population in *tpc2*^*−*^ cells was apparent when cells developed for 4 h were loaded with Lysosensor DND-160 (Fig. 4a, b). Acidic vesicles play an important role in early development as weak bases which neutralize acidic vesicles cause a reduction in the number of aggregates [15]. Consistent with an alkalinization of vesicles, *tpc2*^*−*^ cells showed increased sensitivity to inhibition of aggregation by the weak base imidazole (Fig. 4c). To allow direct comparison, development was carried out on filters, in conditions where no delay in the development of *tpc2*^*−*^ cells is apparent, in the presence of increasing concentrations of imidazole or sodium chloride to control for osmotic effects. Aggregate formation after 12 h of starvation was inhibited in *tpc2*^*−*^ cells by 30 mM imidazole but not in Ax2 cells until 40 mM.

### *Tpc2*^*−*^ cells are defective in cell density sensing during growth

**Fig. 5:**
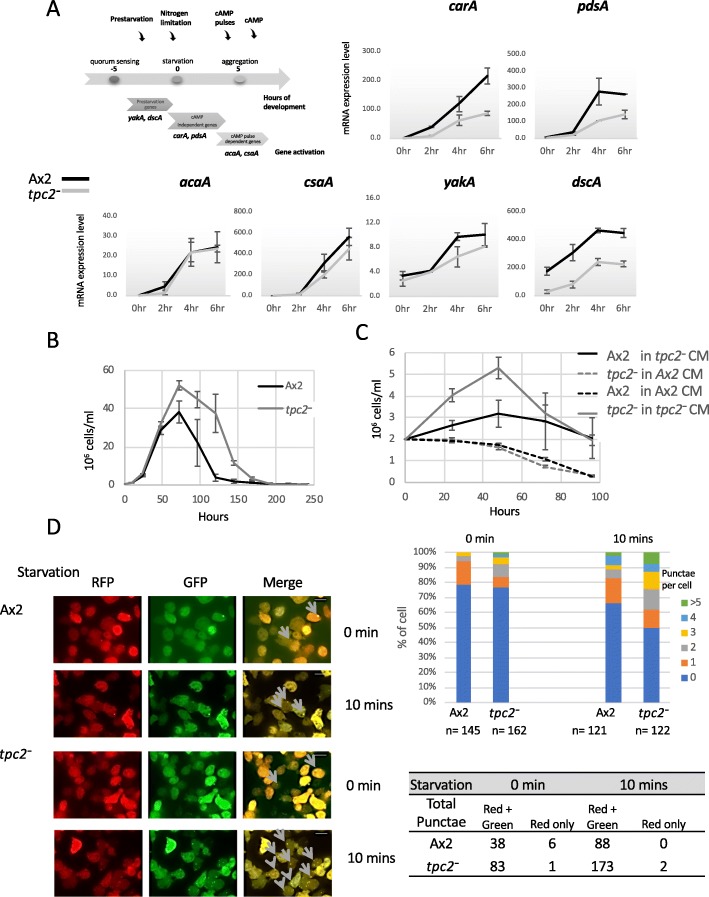
Early developmental responses are altered in *tpc2*^−^ cells. **a** Schematic diagram showing the time course of the expression of selected genes during early *Dictyostelium* development, including prestarvation genes (*yakA* and *dscA*), cAMP pulse-independent genes (*carA* and *pdsA*) and pulse-dependent genes (*acaA* and *csaA*). Ax2 and *tpc2*^−^ cells were harvested from exponential growth, washed twice with HKC-LoCa buffer, resuspended at a density of 1.25 × 10^7^ cells/ml and shaken at 100 rpm at 22 °C for up to 6 h. Every 2 h, 1.4 × 10^7^ cells were sampled for mRNA extraction and gene expression quantified by real time RT-PCR. Results are the average of two independent experiments ± SEM. **b** Ax2 and *tpc2*^−^ cells were seeded at a density of 0.7 × 10^6^ cells/ml in HL5 and grown in shaking suspension at 22 °C. Cells were counted at the ninth hour and then every 24 h after seeding, and the cell number is plotted against the time. Results are the average of three independent experiments ± SEM. **c** Conditioned media were collected by seeding Ax2 and *tpc2*^*−*^ cells at a density of 0.7 × 10^6^ cells/ml in HL5 and growing in shaking suspension at 22 °C for 48 h, and cells were removed by centrifugation. Exponentially growing Ax2 and *tpc2*^*−*^ cells were harvested and resuspended in conditioned media from either *tpc2*^*−*^ or Ax2 cells at a cell density of 2 × 10^6^ cells/ml. Cells were incubated at 22 °C and counted at the times shown. Results are the average of three independent experiments ± SEM. **d** Ax2 and *tpc2*^−^ cells stably expressing the autophagy marker RFP-GFP-Atg8 [33] were imaged either during exponential growth or following 10 min of starvation in HKC-LoCa buffer. Representative punctae are highlighted by arrows. Scale bars, 10 μm. The number of punctae per cell and the number of red/green and red-only punctae were quantified. One experiment representative of three is shown. Further images are shown in Additional file 5: Figure S5

To identify the stage(s) at which loss of TPC2 caused a delay in development, the mRNA levels of a number of early developmental genes were quantified (Fig. 5a). The analysis included genes known to be induced by extracellular pulses of cAMP (*carA*, *pdsA*), genes turned on earlier in development which are independent of the pulses (pulse-independent genes *acaA*, *csaA*) and genes induced prior to the onset of development (*yakA*, *dscA*) (reviewed in [10]). Although reduced expression of pulse-induced genes was apparent, the earliest difference in the expression levels was seen for the *dscA* gene with a difference in the expression apparent in growing cells.

The *dscA* gene is known to be regulated by a number of factors as the cells move towards starvation, including cell density [34], so the growth behaviour of cells in shaken suspension was investigated. Following seeding at low cell density, *tpc2*^*−*^ cells have a similar growth rate to parental cells when grown in shaking suspension (Fig. 5b). However, at a density when Ax2 cells reach stationary phase and cell number peaks, the *tpc2*^*−*^ population carries on increasing in cell number, consistent with a defect in sensing population density and/or nutrient limitation. To determine whether this prolonged growth was due to a factor secreted by *tpc2*^*−*^ cells, conditioned medium (CM) was prepared from both Ax2 and *tpc2*^*−*^ cells following 48 h of growth. Exponentially growing Ax2 and *tpc2*^*−*^ cells were resuspended in CM from both cell types and cell number monitored (Fig. 5c). Ax2 CM supported no increase in cell number in either Ax2 or *tpc2*^*−*^ cells and then a gradual decline from 24 h. However, CM from *tpc2*^*−*^ cells was able to support a continued increase in cell number in Ax2 cells over 48 h, consistent with the presence of a factor that prolonged growth (or the absence of an inhibitory factor). The *tpc2*^*−*^ cells resuspended in *tpc2*^*−*^ CM showed a higher increase in cell number, again peaking at 48 h. This greater increase than seen in Ax2 cells could be due to the continued release of the factor supporting the prolonged growth or could reflect an additional altered sensitivity of the *tpc2*^*−*^ cells to secreted factors.

### *Tpc2*^*−*^ cells can rapidly sense starvation

As differences are apparent in growing *tpc2*^*−*^ cells, it is possible that the delay in early development was due to a failure to rapidly sense starvation. One of the earliest responses reported as the cells enter starvation is an increase in the number of autophagosomes within 10 min, peaking 20–30 min after starvation [35]. To quantify autophagosomes, a construct driving the expression of RFP-GFP-Atg8 was introduced into both Ax2 and *tpc2*^*−*^ cells. This protein associates with autophagosomes allowing them to be quantified by counting fluorescent punctae. Consistent with the changes apparent prior to the onset of development, exponentially growing *tpc2*^*−*^ cells showed a slightly increased number of autophagosomes compared to Ax2 cells (Fig. 5d, Additional file 5: Figure S5). Both Ax2 and *tpc2*^*−*^ cells showed a rapid increase in the number of autophagosomes following 10 min of starvation with a higher number of cells with autophagosomes apparent in *tpc2*^*−*^ cells. In Ax2 cells, only 17% of cells had more than two punctae whereas 38% of *tpc2*^*−*^ cells had at least two detectable punctae 10 min after starvation, with 7% having five or more in contrast to 2% in Ax2. This confirms that some rapid responses to starvation are intact in *tpc2*^*−*^ cells. In other systems, RFP-GFP-Atg8 has been used to measure autophagic flux as the GFP signal is quenched on the fusion of the autophagosome with the lysosome. However, in agreement with other reports using this marker in *Dictyostelium* [36, 37], very few red-only punctae were seen (Fig. 5d, Additional file 5: Figure S5). There were fewer in growing *tpc2*^*−*^ cells compared to Ax2 cells, but the numbers were too small to determine significance.

### The inhibition of mTORC1 activity in response to starvation is compromised in cells deficient in TPC2

**Fig. 6:**
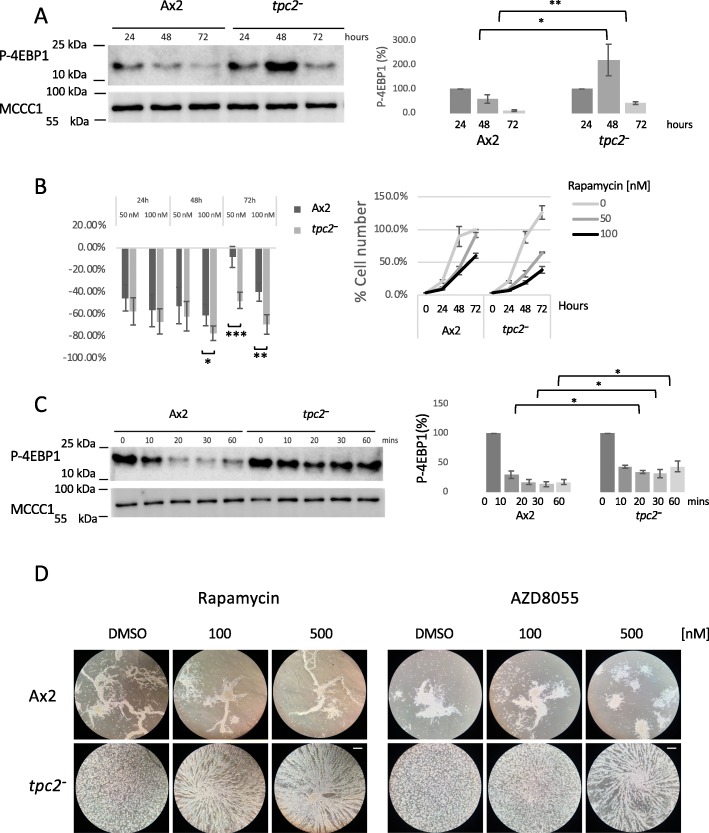
The growth and developmental phenotypes of *tpc2*^−^ cells are dependent on increased mTORC1 activity. **a** Ax2 or *tpc2*^*−*^ cells were grown in shaking suspension as described in the legend of Fig. 5b. Cells were harvested at the times shown. Whole cell lysates were immunoblotted using antibodies against 4E-BP1 phosphorylation (T37/T46). MCCC1 was used as a loading control [41]. One experiment representative of five is shown. The level of 4E-BP1 phosphorylation was quantified with Image Studio Lite (LI-COR), and the average of five independent experiments is shown. **b** Growth of cells was analysed as described (Fig. 5b) but in the presence of the indicated concentrations of rapamycin or DMSO (vehicle control). The percentage inhibition of cell number is shown, Ax2 in black and *tpc2*^−^ in grey. Control Ax2 cell number at 72 h is defined as 100%. Average and SEM of four independent experiments. **c** Exponentially growing parental Ax2 and *tpc2*^−^ cells were harvested and resuspended at a density of 1.4 × 10^7^ cells/ml in HKC buffer and incubated at 22 °C and 120 rpm shaking for the times shown. Whole cell lysates were immunoblotted using antibodies against phosphorylated 4E-BP1. The level of MCCC1 was used as a loading control [41]. One experiment representative of three. The level of 4E-BP1 phosphorylation was quantified with Image Studio Lite (LI-COR), and the average of three independent experiments is shown ± SEM. Statistical analysis was conducted using Student *t* tests. Non-significant, NS; *P* > 0.05; *P ≤ 0.05, **P ≤ 0.01, ***P ≤ 0.001. **d** Ax2 and *tpc2*^−^ cells were developed under buffer as described in the legend of Fig. 2b in the presence of the indicated concentrations of rapamycin or AZD8055. Wells were imaged after 6 h. Scale bars, 200 μm

Mammalian mTORC1 is a protein kinase associated with acidic vesicles, where it has been found in a complex with TPC proteins [20], and has a major role in sensing starvation (reviewed in [38]). To determine if the prolonged growth phase of *tpc2*^*−*^ cells correlated with increased mTORC1 activity, the level of phosphorylated 4E-BP1, a known mTORC1 substrate previously used to monitor mTORC1 activity in *Dictyostelium* [39, 40], was determined by western blot. In parental cells, the level of phosphorylated 4E-BP1 was reduced after 48 h of growth (Fig. 6a) as the cells entered the stationary phase. In *tpc2*^*−*^ cells, the level of phosphorylated 4E-BP1 was increased after 48 h of growth as the cells failed to enter the stationary phase but was reduced at 72 h as the cells entered the stationary phase at higher cell density.

In contrast, no difference in the level of phosphorylation was detected in *tpc2*^*−*^ cells using antisera raised against the mTORC2 substrate SGK1 phosphorylated on Serine 422 (Additional file 6: Figure S6A), recently reported to monitor phosphorylation of an mTOR substrate in *Dictyostelium* [42]. Rapamycin, an inhibitor of mTORC1 activity, inhibits *Dictyostelium* cell growth [43], and *tpc2*^*−*^ cells show an increased sensitivity to rapamycin (Fig. 6b) as 50 nM rapamycin reduces the *tpc2*^*−*^ cell number by 47% at 72 h, but only by 8% for Ax2 cells. This is consistent with the failure to inhibit mTORC1 at the normal density at which Ax2 cells enter the stationary phase driving the prolonged cell proliferation in *tpc2*^*−*^ cells.

Does a delay in reducing mTORC1 activity on starvation explain the delayed aggregation of *tpc2*^*−*^ cells? As previously reported, the phosphorylation of 4E-BP1 decreases within 10 min of starvation of *Dictyostelium* cells (Fig. 6c). This decrease in phosphorylation was also seen in *tpc2*^*−*^ cells, again showing rapid responses to starvation do occur, but the decrease was less pronounced in *tpc2*^*−*^ cells, consistent with a compromised ability to switch off mTORC1 activity on starvation. Antisera raised against the mTORC2 substrate phospho-SGK1 (S422) detected a loss within 10 min of starvation in Ax2 cells, but in *tpc2*^*−*^ cells, no decrease was seen (Additional file 6: Figure S6B). As the levels of both phosphorylated proteins were altered in *tpc2*^*−*^ cells, the ability of either rapamycin or AZD8055 (a dual inhibitor of both mTORC1 and mTORC2 [44]) to rescue the aggregation defect in *tpc2*^*−*^ cells was monitored. Both inhibitors reversed the delay in aggregation (Fig. 6d) with streams apparent following 6 h of starvation, consistent with a reduced ability to switch off mTORC1 in *tpc2*^*−*^ cells contributing to their early developmental phenotype. The concentration of rapamycin required was comparable to those used by others to inhibit mTORC1 in *Dictyostelium* [39, 42], and a similar concentration of AZD8055 to that used in other systems [45] rescues aggregation in *tpc2*^*−*^ cells. Rescue by both compounds is consistent with mTORC1 being the relevant target.

## Discussion

Here, we define a role for the TPC2 protein in early development in response to starvation in *Dictyostelium* as disruption of the *tpc2* gene causes a delay in early development on an agarose matrix, with large aggregation streams apparent. This delay is exacerbated when aggregates are allowed to form under buffer but is not apparent when cells are developed on filters suggesting that the extent of the delay is dependent on environmental conditions. Despite any early delay, development is completed to generate apparently normal fruiting bodies. Partial, but not complete, rescue of development of *tpc2*^*−*^ cells by exogenous cAMP pulsing suggests reduced efficiency of both production of cAMP and response to it.

In mammalian cells, TPC2 proteins have been shown to facilitate Ca^2+^ release from acidic stores. During early *Dictyostelium* development, extracellular cAMP triggers Ca^2+^ influx from the extracellular medium (measured by uptake of radiolabelled Ca^2+^) and changes in cytosolic Ca^2+^, both of which are not detectable in *iplA*-null cells [12, 46]. Changes in cytosolic Ca^2+^ in *iplA*^*−*^ cells were measured using YC-nano60 [12], a related but distinct genetically encoded Ca^2+^ indicator to the YC-nano15 used here. YC-nano60 has a lower affinity for Ca^2+^ than YC-nano15 [28], so there could be small changes in cytosolic Ca^2+^ in *iplA*^*−*^ cells mediated by the channels such as TPC2 or TrpML channels, below the level detected by YC-nano60. However, it is clear that the majority of the cytosolic Ca^2+^ response normally seen in response to extracellular cAMP is dependent on IplA channels and not on TPC2, although the rise in cytosolic Ca^2+^ is delayed in *tpc2*^*−*^ cells. The slight dampening of the response could be directly due to the loss of the TPC2 protein as it has been hypothesized that small Ca^2+^ responses from acidic stores act to prime receptors such as the IP_3_ receptor to facilitate larger Ca^2+^ release from neutral stores [13].

The increased rate of Ca^2+^ uptake of isolated vesicles in vitro could suggest that TPC2 channels normally act as Ca^2+^ leak channels which are absent in the vesicles from *tpc2*^*−*^ cells or that loss of TPC2 leads to an alteration in the level or activity of Ca^2+^ pumps found in these vesicles. The increased expression of *patA* mRNA, if translated into an increased expression of the whole complex at the protein level, could explain this as it acts as a Ca^2+^ pump found on acidic compartments. The *patA* gene is known to be regulated by Ca^2+^ levels [29], so these changes could be part of a cellular response to the reduced cytosolic Ca^2+^ levels seen in *tpc2*^*−*^ cells. In *Dictyostelium*, arachidonic acid is the only agent, other than Ca^2+^ itself, known to trigger Ca^2+^ release from these preparations which contain vesicles derived from both acidic and neutral stores [30], and TPC2 is not required for arachidonate-induced release. Arachidonic acid is not found in *Dictyostelium* [47], but phospholipase A has been shown to be required for the aggregation in the absence of PI-3 kinase activity [48], so a related lipid could be an endogenous trigger for Ca^2+^ release.

In vivo, the overall pH of the acidic vesicle population was increased in *tpc2*^*−*^ cells at 4 h of starvation. pH-dependent lysosomal dyes such as the one used here have been reported to be of limited use in *Dictyostelium* due to the very low pH of the vesicles [31]. Despite this, increased vesicle pH is detected in *tpc2*^*−*^ cells, suggesting that the change in pH can be sensed by the probe. Alkalinization of acidic vesicles by depletion of TPC2 has also been reported in melanocytes [49]. However, in a human megakaryocytic cell line, MEG-01, loss of TPC2 resulted in vesicle acidification [50], and in mouse embryo fibroblasts, loss of TPC proteins does not affect lysosomal pH [6]; so, the consequence of TPC2 loss on acidic vesicle pH is cell type specific. Acidic stores have an important role during early development in *Dictyostelium* [14]. Weak bases enter acidic compartments where they become protonated and trapped, neutralizing them, and this leads to reduced aggregation in *Dictyostelium* development [15].The increased sensitivity of *tpc2*^*−*^ cells to the inhibition of aggregation by weak bases is consistent with the less acidic nature of the stores in *tpc2*^*−*^ cells requiring lower concentrations of weak base to neutralize.

The reduced expression of *dscA* already apparent in exponentially growing *tpc2*^*−*^ cells points to defects in density sensing factors active before the onset of starvation [34]. The prolonged growth in of *tpc2*^*−*^ cells in culture and the ability of conditioned medium from *tpc2*^*−*^ cells to support an increased cell density of Ax2 cells compared to Ax2-derived conditioned medium supports this. A number of factors have been identified which regulate *dscA* expression including prestarvation factor (PSF) and conditioned medium factor (CMF) [51, 52]. Growing cells continuously secrete PSF and monitor the ratio of PSF to bacterial food source [53]. When the bacterial titre falls, PSF induces expression of genes such as *dscA* and *yakA* [54], and the expression of *dscA* is reduced in *tpc2*^*−*^ cells, although the expression of *yakA* is not much altered, suggesting that production of, or response to, PSF might not explain the phenotype of *tpc2*^*−*^ cells. On starvation, PSF levels fall and CMF production is induced [55]. Cells that fail to accumulate CMF fail to aggregate through a failure to activate the cAR1 cAMP receptor [56]. In addition, the secreted proteins AprA and CfaD function as the reporters of cell density, and disruption of the gene encoding either of these leads to increased cell density at stationary phase [57, 58], similar to *tpc2*^*−*^ cells, leading to a simple hypothesis that these proteins work through the regulation of TPC2 channels in *Dictyostelium* to sense cell density. However, unlike *tpc2*^*−*^ cells, *aprA*^*−*^ and *cfaD*^*−*^ cells show an increased growth rate during exponential growth.

Cells deficient in TPC2 can rapidly sense starvation as within 10 min of removal of growth media they have reduced levels of the phosphorylated form of the mTORC1 substrate 4E-BP1 and an increased number of autophagic vesicles. In mammalian cells, rapamycin-induced mTORC1 inhibition leads to increased autophagy via inhibition of phosphorylation of Atg1. However, in *Dictyostelium*, short-term rapamycin treatment does not induce autophagy [36], so there could be an alternative mechanism to regulate autophagy at the onset of starvation, although downregulation of mTORC1 activity correlates with increased autophagy induced by *Mycobacterium marinum* infection [40]. The increased number of autophagic vesicles detected in *tpc2*^*−*^ cells could reflect increased generation of autophagosomes or could reflect reduced flux and further processing. Once formed, autophagosomes fuse with lysosomes, but the altered pH of lysosomes seen in *tpc2*^*−*^ cells could mean that this fusion, or further flux through the system, is impaired. In other systems, the RFP-GFP-Atg8 protein has been used to monitor flux as the GFP signal is quenched by the reduced pH when the autophagosome fuses with the lysosome so the red only punctae mark autolysosomes [33]. However, in *Dictyostelium* cells (strain Ax4) expressing this marker, no RFP-only punctae have been seen unless the acidic vesicle pH is increased by incubation with weak bases, suggesting that in the absence of weak base, the pH may be low enough to quench even the RFP signal [36]. There is a single report suggesting a very low number of RFP-only punctae in Ax2 cells [37]. The low number we see here in Ax2 cells is consistent with these observations. Although the numbers are very small, a reduced number of RFP-only punctae are apparent in growing *tpc2*^*−*^ cells, compared to Ax2 cells. Further analysis is required to determine if this reflects differential flux.

mTORC1 is found associated with lysosomes and late endosomes in mammalian cells and with the vacuolar membrane in yeast [59, 60], and components of the mTORC1 complex have been found on bacteria-containing vacuoles in *Dictyostelium* which have characteristics of post-lysosomes [40]. Mammalian mTORC1 interacts with TPC proteins, and recently rapamycin has been shown to induce lysosomal Ca^2+^ release in a TPC-dependent manner [20, 21, 61], consistent with mTORC1 activity inhibiting TPC channel function. In *Dictyostelium*, long-term treatment with rapamycin (48–60 h) leads to increased cytosolic Ca^2+^ levels [43]. We show a reciprocal relationship between higher levels of a phosphorylated mTORC1 substrate in cells lacking TPC proteins, both during growth and immediately following starvation. This correlates with an increased density at the stationary phase in liquid culture. Unfortunately, no antisera are available to monitor total 4E-BP1 levels in *Dictyostelium*, so we cannot rule out that the changes in amount of phosphorylated protein could reflect a change in total 4E-BP1 levels, although such changes have not been reported in other systems and RNAseq data suggests an increase in mRNA levels during the first hour of development [62]. In mammalian cells, SGK1 is a substrate for mTORC2 [63]. In contrast to 4E-BP1 phosphorylation, phosphorylation events detected by an anti-phospho-SGK1 (Ser422) antiserum were no different between Ax2 and *tpc2*^*−*^ cells during prolonged growth in culture. Rapamycin inhibits cell growth in *Dictyostelium* [43] and *tpc2*^*−*^ cells show increased sensitivity to this inhibition consistent with increased mTORC1 activity in these cells. Similarly when *tpc2*^*−*^ cells are starved, although the level of phosphorylated 4E-BP1 is reduced, it remains at a higher level than in Ax2 cells and the delay in the development of *tpc2*^*−*^ cells is reduced in the presence of the mTORC1 inhibitor rapamycin consistent with this increased activity being at least partially responsible for the delay. Rapamycin treatment of *Dictyostelium* has recently been shown to induce genes required for the growth development transition including *carA*, consistent with their reduced expression in *tpc2*^*−*^ cells [42].

## Conclusion

Loss of TPC2 protein in *Dictyostelium* prolongs growth in culture and delays the onset of development. The *tpc2*^*−*^ cells have altered kinetics of calcium signalling and increased pH of acidic vesicles. The null cells have increased phosphorylation levels of the mTORC1 substrate 4E-BP1 following starvation, and the growth and developmental phenotype can be rescued by mTORC1 inhibition, consistent with the phenotypic consequences of TPC2 loss being mediated through mTORC1 activity, a novel pathway to regulate mTORC1 activity.

## Methods

### *Dictyostelium* growth and development

*Dictyostelium discoideum* strain Ax2 and derivatives were grown on SM agar plates in association with *Klebsiella aerogenes* or axenically in HL5 media (Formedium, UK) in shaking suspension at 220 rpm at 22 °C. Cells were harvested during exponential growth.

For filter development, harvested cells were washed twice with LPS (25 mM KCl, 0.5 g/ml streptomycin sulphate, 2.5 mM MgCl_2_, 27 mM NaH_2_PO_4_, 24 mM Na_2_HPO_4_ pH 7), resuspended in buffer at a density of 5 × 10^7^ cells/ml and 100 μl placed on boiled nitrocellulose filters (0.45 μm; Millipore Limited), supported on LPS-soaked blotting paper. Similarly, for agarose and under buffer development, cells were washed twice with appropriate buffer and 3.84 × 10^6^ cells plated either on 1% agarose in HKC-LoCa buffer (10 mM HEPES, 10 mM KCl, 250 μM Ca^2+^, pH 7.0) or directly on the plate surface in a 6-well plate, final cell density 4 × 10^5^ cells/cm^2^, incubated at 22 °C for the appropriate time. In fluorescence experiments, a 35-mm imaging dish with an ibidi standard base and low walls was used for development.

Conditioned medium was collected by seeding Ax2 and *tpc2*^*−*^ cells at a density of 0.7 × 10^6^ cells/ml in HL5 and growing in shaking suspension at 22 °C, 220 rpm for 48 h. After 48 h, cells were removed by two rounds of centrifugation and the supernatant was taken as a conditioned medium.

### Generation of *tpc2*^*−*^ cells

A plasmid was designed to disrupt the *tpc2* gene by homologous recombination to replace the central coding region with a blasticidin resistance cassette as described in [25]. The strategy inserts a blasticidin resistance cassette in the coding sequence after amino acid 216 and replaces the coding sequence until amino acid 1645. This removes all the predicted transmembrane domains. A 5′ arm from 21 to 650 of the *tpc2* coding sequence and 3′ arm (positions 4934 to 6051 of the *tpc2* genomic sequence) were PCR amplified and inserted either side of the blasticidin resistance cassette in pLPBLP [64] to generate pLPBLP-*tpc*. This was digested with KpnI and BamHI and electroporated into Ax2 cells. Following selection with 10 μg/ml blasticidin, individual resistant colonies were screened for disruption of the *tpc2* gene by PCR and loss of expression confirmed by RT-PCR (Additional file 1: Figure S1). All phenotypes were verified in three disruption strains generated in independent transformation experiments. Data shown here is for clone TPC22B20. Attempts to clone the full-length coding sequences for complementation of the *tpc2*^*−*^ cells was not successful, probably due to the presence of large numbers of AAC repeat sequences within the gene.

### Vesicle preparation

Adapted from Malchow et al. [65], vegetatively growing cells were washed twice with KK_2_ buffer (19 mM KH_2_PO_4_, 3.6 mM K_2_HPO_4_); 3.5 × 10^8^ cells were resuspended at a density of 1.4 × 10^7^ cells/ml in KK_2_ plus 5 mM EGTA and shaken at 120 rpm for 4 h, pulsing with exogenous cAMP (50 nM every 6 min). Cells were washed with ice-cold 20 mM HEPES, pH 7.2, twice and resuspended in 4 ml of this buffer containing 1× proteinase inhibitor; Roche. Samples were disaggregated using 21-G needles then homogenized by passage through 3-μm nucleopore filters (Millipore). Homogenate was added to 4 ml of 2× buffer 1 (100 mM KCl, 2 mM MgCl_2_, 6% sucrose, 2× proteinase inhibitor, Roche). After centrifugation for 5 min at 200*g* to remove residual intact cells, P0 was obtained by centrifugation at 3800*g* for 5 min and resuspended in 30 μl buffer 2 (10 mM Tris HCl, 50 mM KCl, 1 mM MgCl_2_, 3% sucrose, 2× proteinase inhibitor). P1 was obtained by centrifugation at 12,000*g* for 20 min and resuspended in 25 μl of buffer 2. P1 vesicles contained a mixture of markers of both the endoplasmic reticulum (calnexin) and acidic contractile vacuole (dajumin). All the experiments described here were performed on P1 vesicles. All procedures were carried out at 4 °C. Samples were either used fresh or aliquots snap frozen on dry ice and stored at − 80 °C up to 7 days.

### Ca^2+^ uptake and release in vitro

Vesicles (2–400 μg protein) were added to 100 μl ice-cold Ca^2+^ uptake buffer (10 mM HEPES, 50 mM KCl, 2 mM MgCl_2_, 3% sucrose) containing 100 nM NaN_3_, 6.6 μM Fluo-3 (Sigma Aldrich, UK), 0.4 mM Mg^2+^-ATP and 12 μg/ml oligomycin. Free ionized Ca^2+^ was measured via Fluo-3 (490 nm excitation and 535 nm emission) in a 96-well plate, using a fluorometer (NovoStar).

### Fluorescence microscopy for pH indicator

Cells were grown in LoFlo medium to reduce auto-fluorescence and allowed to develop under Lower Pad Solution (LPS: 1.5 g KCl, 0.5 g MgCl_2_.6H_2_O, 3.75 g NaH_2_PO_4_, 3.375 g Na_2_HPO_4_ per litre pH 7.0) for 4 h before staining with a pH-sensitive indicator LysoSensor Yellow/Blue DND-160 (PDMPO), a dye for measuring the pH of acidic organelles with a pKa of 4.2 (Thermo Fisher Scientific) at final concentration of 2 μM on a 35-mm imaging dish (ibidi μ-Dish) for 15 min. The blue and green channel images and ratio images were acquired by using a widefield fluorescence inverted microscope (DeltaVision™ Elite) with two emission images at 438/48 nm and 525/48 nm, both excited at 390/18 nm.

### Fluorescence microscopy for autophagy

Cells were transformed with plasmid vectors expressing the autophagic marker RFP-GFP-Atg8 [33], from the *Dictyostelium* Stock Centre (http://dictybase.org/) via electroporation, with selection with 10 μg/ml G418 (Invitrogen). To induce autophagy via starvation, vegetative cells were washed three times with LPS buffer then allowed to attach to a 35-mm ibidi μ-Dish for 10 min. Cells incubated in growth medium served as the non-starvation control. The autophagic flux was observed using an Olympus IX71 inverted phase/fluorescence microscope with a Hamamatsu ORCA-R2/C10600-10B digital CCD camera. The GFP and RFP images were captured with FITC and TRITC filters, respectively.

### Fluorescence microscopy for calcium indicator

Cells were transformed with plasmid vectors to drive expression of the ultrasensitive Ca^2+^ indicator YC-Nano 15, kindly provided by Prof Takeharu Nagai [28], [66] via electroporation with selection by 10 μg/ml G418. Resulting cells were washed twice with HKC buffer containing 0.25 mM Ca^2+^, before plating on a 35-mm imaging dish. The Ca^2+^ changes were acquired using a widefield fluorescence inverted microscope (DeltaVision™ Elite) at a wavelength of 438/24 nm (CFP) excitation and emitted fluorescence captured simultaneously at 548/22 nm (YFP) and 475/24 nm (CFP) every 15 s with an EMCCD camera.

The image stack in .dv format was imported into FIJI 1.52b (https://imagej.net/Fiji/Downloads). The image calculator function in FIJI was used to calculate the YFP to CFP emission signal ratio. Cells were selected and tracked throughout the frames of the image using the FIJI plugin Trackmate v3.5.3 which sorts cells into tracks based on their movement in the subsequent frames [67]. Cell detection was performed using a Laplacian of Gaussian filter with 16 μm size and median filter. The detection threshold was defined manually based on individual trials. The Simple LAP tracker was used to calculate cell trajectories. A maximum distance for frame-to-frame linking of 12 μm and maximum allowed gap size of two frames were selected. Data was preprocessed using MATLAB R2015a. Tracks containing less than half of the intensity data over the course of the imaging, or over the course of the mean baseline ratio (defined as the frames before cAMP was added), were discarded. Normalization was performed by subtracting the mean signal intensity at the baseline period from the time series and dividing by the baseline. The percent change in intensity after addition of cAMP was calculated for each cell by dividing the difference of the maximum response to the baseline by the baseline. The mean and standard error for each trial were calculated from the response change over each cell in the trial.

### Western blot analysis

Western blotting was performed using PVDF membrane and either anti-phospho-4E-BP1 (Thr37/46) (Cell Signaling Technology #9459), diluted 1:1000 [39] or anti-phospho SGK1 (Ser422) (Abcam ab55281) diluted 1:500 [42] at 4 °C, overnight. The secondary antibody was polyclonal goat-anti-rabbit HRP, 1:5000 at room temperature for 1 h. Super Signal Western Femto (Thermo Scientific) substrate was used for chemiluminescence. The loading control MCCC1 [41] was detected using Alexa 680-conjugated streptavidin, diluted 1:1000. Blots were visualized on the Odyssey Fc imaging system (LI-COR, Lincoln).

### Quantitative real-time RT-PCR

Total RNA was isolated using TRIzol® Reagent (Life Technologies), from which 10 μg was treated with TURBO DNA-free™ Kit (Life Technologies) to remove residual gDNA. Two micrograms DNase-treated total RNA was reverse transcribed to cDNA using SuperScript™ IV First-Strand Synthesis System (Invitrogen). qRT-PCR was performed with 100-fold diluted cDNA and the SensiFAST SYBR No-ROX Kit (Bioline) in a Rotor-Gene thermocycler (Corbett Research) using *ig7* for normalization [68]. All primers (Table 1) were designed using Primer-Blast (https://www.ncbi.nlm.nih.gov/tools/primer-blast/), except for *acaA* [68, 69] and *yakA* [70].

**Table 1:** Primers used in qRT-PCR to quantify gene expression

Gene	Forward primer	Reverse primer
*Ig7*	TCGATCAGAGACGCAAGTCG	CACCCCAACCCTTGGAAACT
*yakA*	GCACTCGGTCAAAGTCCATC	AATTTGGTGTGGCAACTGGTG
*carA*	GCTGTCAATGGTGGTTTCCC	CTAATTGCAAGACATAAAGTCCACA
*pdsA*	TCCATCCTCTGTCGCTTGTG	CTCTTGGACGGAGATGACCA
*dscA*	AAGGTTTAGTTCAACTCCTCGC	ACACCAAGCTTCTGAACCATCA
*acaA*	GGAGAAAATGTCTGATTTCGCTT	CATTCTAGAGGCGGTATTGGC
*csaA*	AACCATGGGAACCTCAAGCC	TGTGAGGTGCTTGAGTGACA
*patA*	TACAAACGATGGTCCTGCCC	ACGGCACGAACAATTGAAGC

### Statistical and bioinformatic analysis

Statistical comparisons were conducted using Student *t* tests (two-tailed) or Wilcoxon rank-sum test by using Microsoft Excel or R (https://www.r-project.org/). Protein sequence analysis was conducted by using BioEdit (http://www.mbio.ncsu.edu/bioedit/bioedit.html) and NCBI Blast (https://blast.ncbi.nlm.nih.gov/Blast.cgi).

## Supplementary information

**Additional file 1: Figure S1.** Disruption of the *tpc2* gene. A. Disruption construct. 5’ and 3’ arms were amplified from genomic DNA using primers with restriction enzyme sites compatible with the vector pLPBLP. B. Schematic representation of the homologous recombination event. C. Following transfection into Ax2 cells and selection with Blasticidin, genomic DNA (gDNA) was prepared from single resistant colonies and used in a diagnostic PCR screen to confirm integration of the bsR cassette at the *tpc* locus. Two primer sets (W3, 5’ BSR-LOX and 3’ BSR-LOX, W5) were used in a PCR reaction to check for targeted insertion of the bsR cassette at the 5’ and 3’ ends, respectively. A primer set (W3 and TPC-5-R) was used as the positive control reaction. A random insertion of the Blasticidin resistance (bsR) cassette produced no PCR product for the screen, only targeted insertion of both arms produced 938 bp and 1,271bp bands. Clones T-II-1, T-II-23, T-I-10, T-I-16 and T-I-22 had successful insertions of both the 5’ arm and 3’arm. The PCR bands were resolved on 1% agarose gels. D. Loss of the *tpc2* coding sequence was confirmed by PCR using primers from the central portion of *tpc2* which was replaced by the Bsr cassette. Amplification using primers specific for *apl* and *mcln* genes are used as a control.

**Additional file 2: Figure S2.** Confirmation that YC-nano-15 senses cytosolic Ca^2+^ levels. Ax2 cells expressing YC-nano15 were treated with ionomycin (20 mM), a Ca^2+^ ionophore, in the presence of either 20 mM CaCl_2_ or EGTA for 15 min [19]. The [Ca^2+^]c is detected by FRET which is observed as the ratio of YFP/CFP emission. During incubation with ionomycin and EGTA, the ratio reduced and remained low for the duration of the experiment (15 min, data not shown). In the presence of high Ca^2+^, the ratio increased and peaked within 7.5 minutes, remaining high for the duration of the experiment (15 min, data not shown). These data demonstrate that the YFP/CFP emission ratio of YC-Nano15 is responding to changes of intracellular Ca^2+^ as expected. Scale bar, 50 μm.

**Additional file 3: Figure S3.** Cytosolic Ca2+ in growing Ax2 and *tpc2*^*−*^ cells. Single clones of parental Ax2 or *tpc2*^*−*^ cells expressing YC-nano15 were harvested from exponential growth. The YFP/CFP ratio was imaged using a widefield fluorescence inverted microscope. Values for individual cells are shown.

**Additional file 4: Figure S4.** Arachidonic acid induced Ca^2+^ release from vesicles derived from Ax2 and *tpc2*^*−*^ cells. Vesicles were isolated from Ax2 and *tpc2*^−^ cells developed in shaking suspension in KK2 and EGTA for 4 hours, with exogenous pulses of cAMP (50 nM) every 6 minutes. 200 μg of the vesicles were added to 100 μl Ca^2+^ uptake buffer, including 6.6 μM Fluo-3 as Ca^2+^ indicator, and supplemented with 100 μM NaN_3_, 1.5 mM ATP and 6 μg/ml oligomycin A. Free ionized Ca^2+^ was measured via Fluo-3 (490 nm excitation and 535 nm emission). Representative trace showing calcium release in response to 10 μM arachidonic acid (AA) or ethanol vehicle control (EtOH), one of three.

**Additional file 5: Figure S5.** Further images of cells expressing RFP-GFP-Atg8. Ax2 and *tpc2*^−^ cells stably expressing the autophagy marker RFP-GFP-Atg8 were imaged either during exponential growth (A) or following 10 minutes of starvation in HKC-LoCa buffer (B) as described in the legend to Fig. 5c. Six images from one experiment representative of 3 is shown.

**Additional file 6: Figure S6.** Western blot analysis of Ax2 and *tpc2*^*−*^ cells using anti-phospho SGK1 (Ser 422) as an mTOR substrate. A. Ax2 and *tpc2*^−^ cells were seeded at a density of 0.7 x 10^7^ cells/ml in HL5 and grown in shaking suspension at 22^0^C. Cells were harvested at the times shown. Whole cell lysates were immunoblotted using antibodies against phospho-SGK1 Ser422. MCCC1 was used as a loading control . One experiment representative of three is shown. The level of phosphorylated protein was quantified with Image Studio Lite (LI-COR) and the average of three independent experiments is shown. B. Exponentially growing parental Ax2 and *tpc2*^−^ cells were harvested and resuspended at a density of 1.4 x 10^7^ cells/ml in HKC buffer and incubated at 220C and 120 rpm shaking for the times shown. Whole cell lysates were immunoblotted using antibodies against phospho-SGK1 Ser422. The level of MCCC1 was used as a loading control. One experiment representative of three is shown. The level of phosphorylated protein was quantified with Image Studio Lite (LI-COR) and the average of three independent experiments is shown. Statistical analysis for values at 10, 20 and 30 minutes relative to 0 minutes was conducted using Student *t*-test. ** *P* ≤ 0.01.

## Data Availability

All data generated or analysed during this study are included in this published article [and its supplementary information files].
