# Supplementary figures and images for: A two-pore channel protein required for regulating mTORC1 activity on starvation
Source: BMC Biol. 2020 Jan 22;18:8. doi: 10.1186/s12915-019-0735-4 (PMC6977259; doi:10.1186/s12915-019-0735-4)

A

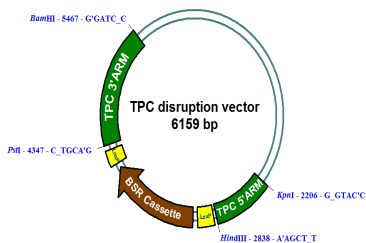

B

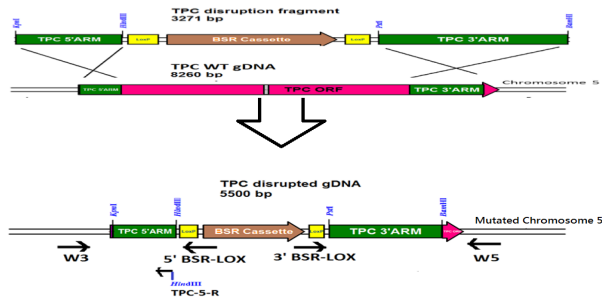

C

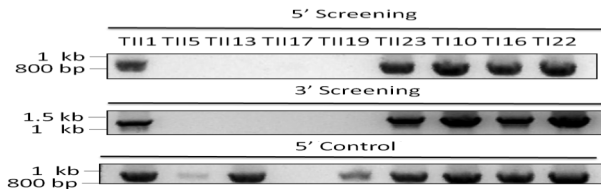

D

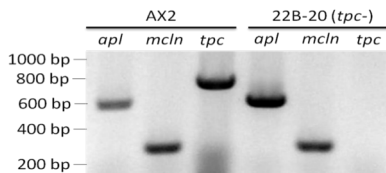

Supplement: Supplementary file 1 — Additional file 1: Figure S1. Disruption of the tpc2 gene. A. Disruption construct. 5’ and 3’ arms were amplified from genomic DNA using primers with restriction enzyme sites compatible with the vector pLPBLP. B. Schematic representation of the homologous recombination event. C. Following transfection into Ax2 cells and selection with Blasticidin, genomic DNA (gDNA) was prepared from single resistant colonies and used in a diagnostic PCR screen to confirm integration of the bsR cassette at the tpc locus. Two primer sets (W3, 5’ BSR-LOX and 3’ BSR-LOX, W5) were used in a PCR reaction to check for targeted insertion of the bsR cassette at the 5’ and 3’ ends, respectively. A primer set (W3 and TPC-5-R) was used as the positive control reaction. A random insertion of the Blasticidin resistance (bsR) cassette produced no PCR product for the screen, only targeted insertion of both arms produced 938 bp and 1,271bp bands. Clones T-II-1, T-II-23, T-I-10, T-I-16 and T-I-22 had successful insertions of both the 5’ arm and 3’arm. The PCR bands were resolved on 1% agarose gels. D. Loss of the tpc2 coding sequence was confirmed by PCR using primers from the central portion of tpc2 which was replaced by the Bsr cassette. Amplification using primers specific for apl and mcln genes are used as a control. [file 12915_2019_735_MOESM1_ESM.pdf]

-40 sec

425 sec

905 sec

Ionomycin+  
 $\text{Ca}^{2+}$

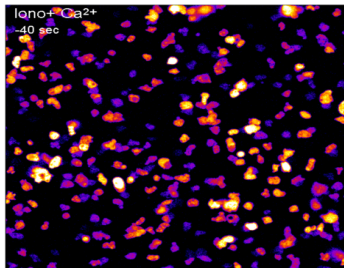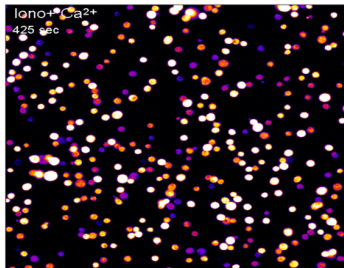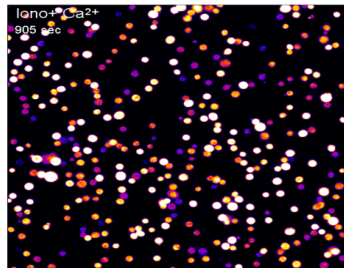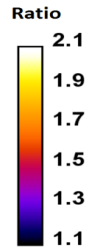

Ionomycin+  
EGTA

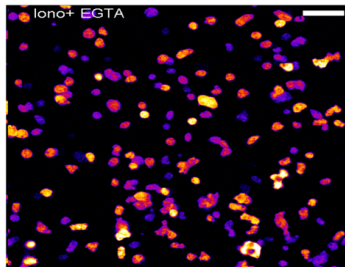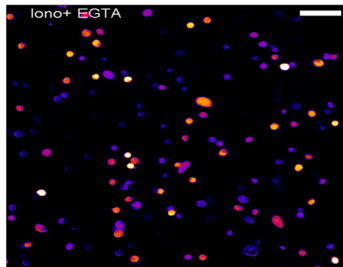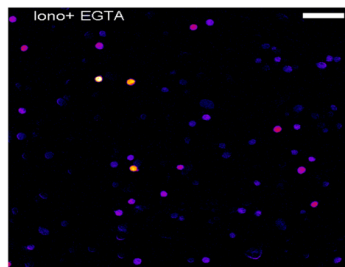

Supplement: Supplementary file 2 — Additional file 2: Figure S2. Confirmation that YC-nano-15 senses cytosolic Ca2+ levels. Ax2 cells expressing YC-nano15 were treated with ionomycin (20 mM), a Ca2+ ionophore, in the presence of either 20 mM CaCl2 or EGTA for 15 min [19]. The [Ca2+]c is detected by FRET which is observed as the ratio of YFP/CFP emission. During incubation with ionomycin and EGTA, the ratio reduced and remained low for the duration of the experiment (15 min, data not shown). In the presence of high Ca2+, the ratio increased and peaked within 7.5 minutes, remaining high for the duration of the experiment (15 min, data not shown). These data demonstrate that the YFP/CFP emission ratio of YC-Nano15 is responding to changes of intracellular Ca2+ as expected. Scale bar, 50 μm. [file 12915_2019_735_MOESM2_ESM.pdf]

Ax2

*tpc2*<sup>-</sup>

YFP/CFP Ratio

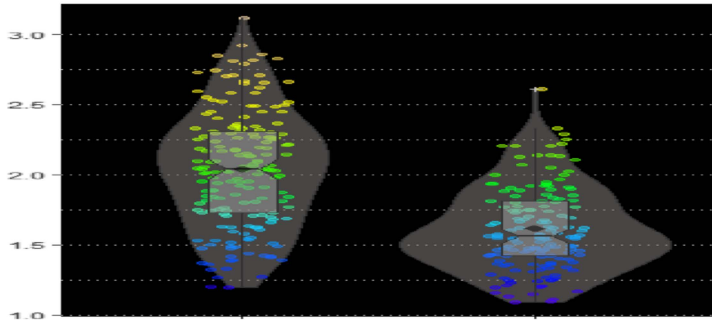

Mean ratio=  $2.04 \pm 0.41$

N= 181

Mean ratio=  $1.62 \pm 0.29$

N=157

Supplement: Supplementary file 3 — Additional file 3: Figure S3. Cytosolic Ca2+ in growing Ax2 and tpc2− cells. Single clones of parental Ax2 or tpc2− cells expressing YC-nano15 were harvested from exponential growth. The YFP/CFP ratio was imaged using a widefield fluorescence inverted microscope. Values for individual cells are shown. [file 12915_2019_735_MOESM3_ESM.pdf]

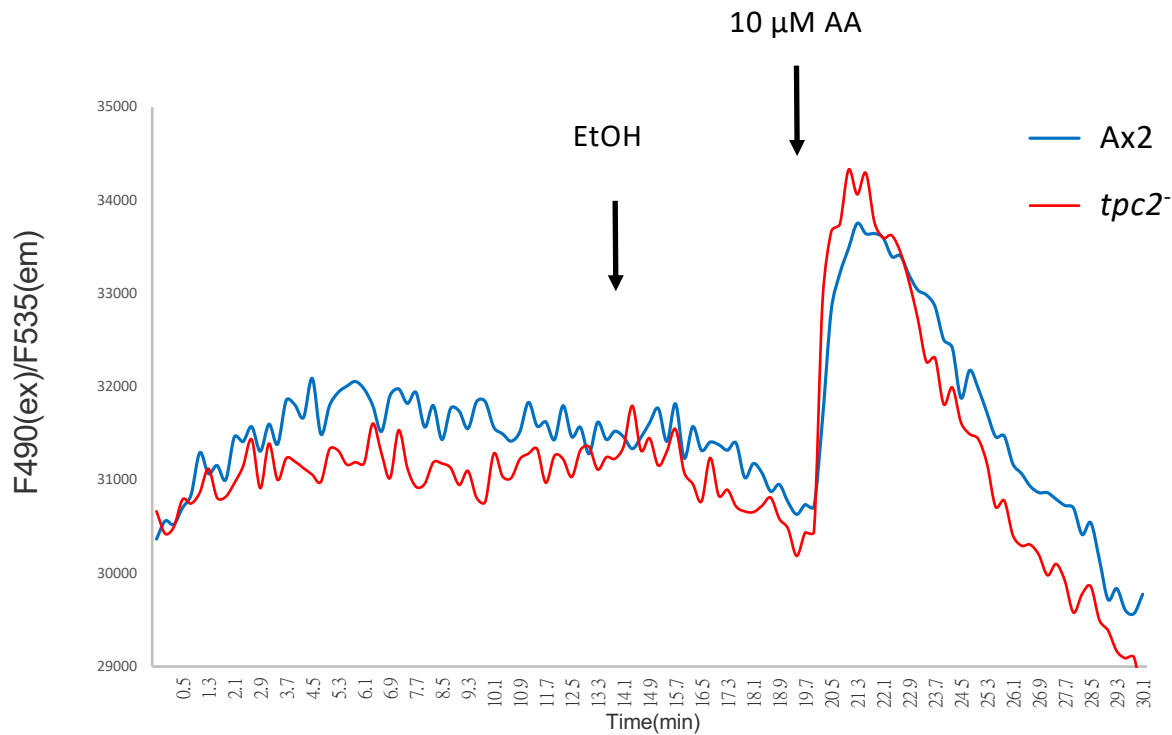

Supplement: Supplementary file 4 — Additional file 4: Figure S4. Arachidonic acid induced Ca2+ release from vesicles derived from Ax2 and tpc2− cells. Vesicles were isolated from Ax2 and tpc2− cells developed in shaking suspension in KK2 and EGTA for 4 hours, with exogenous pulses of cAMP (50 nM) every 6 minutes. 200 μg of the vesicles were added to 100 μl Ca2+ uptake buffer, including 6.6 μM Fluo-3 as Ca2+ indicator, and supplemented with 100 μM NaN3, 1.5 mM ATP and 6 μg/ml oligomycin A. Free ionized Ca2+ was measured via Fluo-3 (490 nm excitation and 535 nm emission). Representative trace showing calcium release in response to 10 μM arachidonic acid (AA) or ethanol vehicle control (EtOH), one of three. [file 12915_2019_735_MOESM4_ESM.pdf]

**A**

0 min

Ax2

*tpc2<sup>-</sup>*

RFP

GFP

Merge

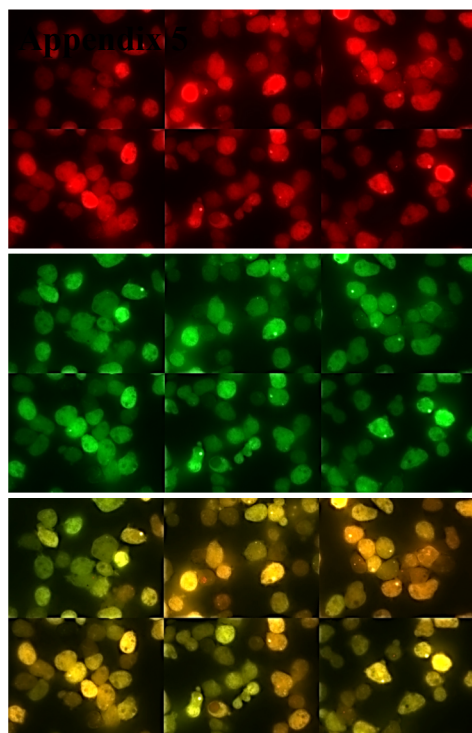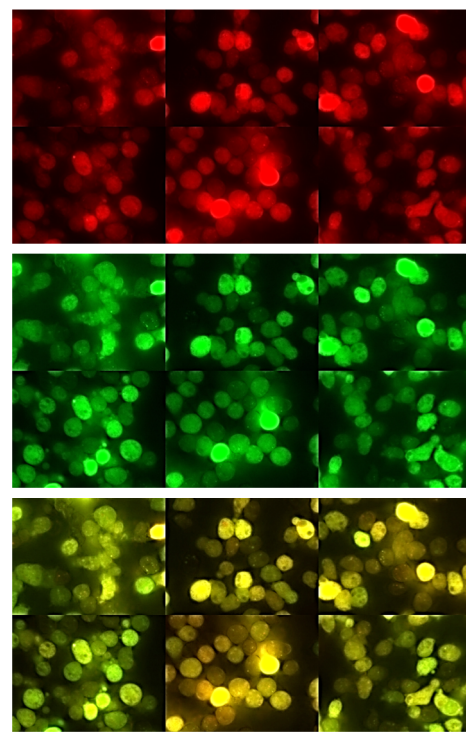**B.**

10 min

Ax2

*tpc2<sup>-</sup>*

RFP

GFP

Merge

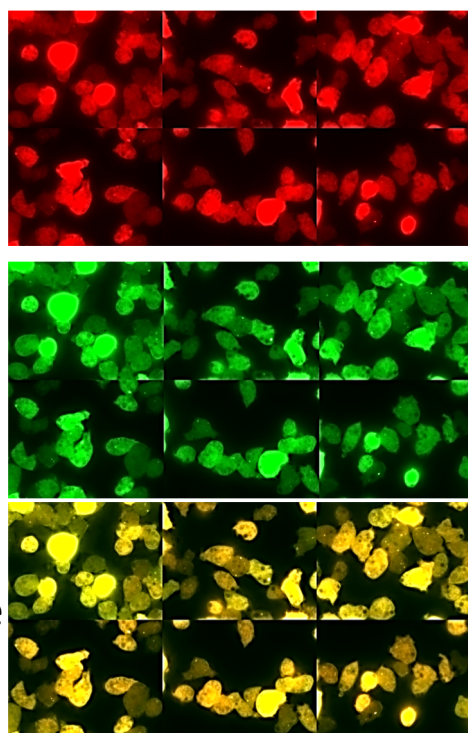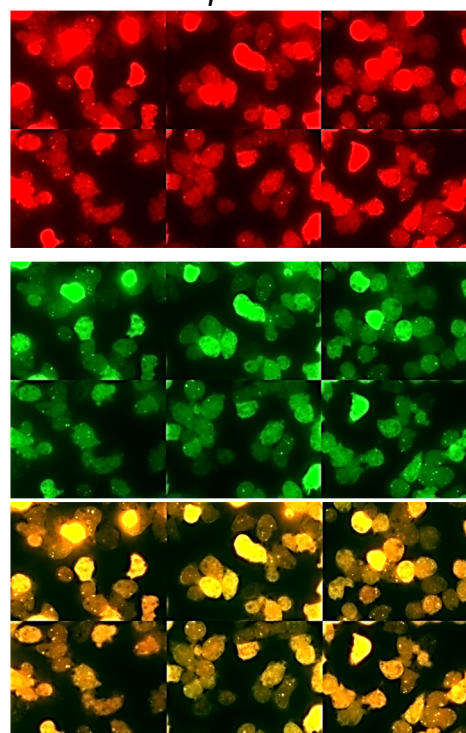

Supplement: Supplementary file 5 — Additional file 5: Figure S5. Further images of cells expressing RFP-GFP-Atg8. Ax2 and tpc2− cells stably expressing the autophagy marker RFP-GFP-Atg8 were imaged either during exponential growth (A) or following 10 minutes of starvation in HKC-LoCa buffer (B) as described in the legend to Fig. 5c. Six images from one experiment representative of 3 is shown. [file 12915_2019_735_MOESM5_ESM.pdf]

A.

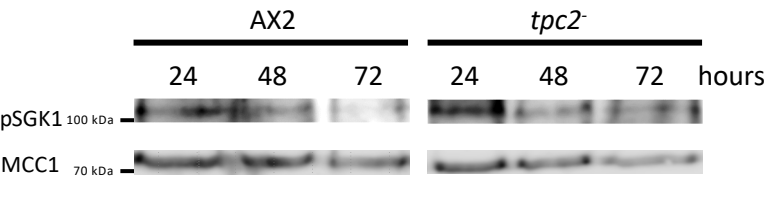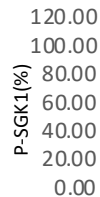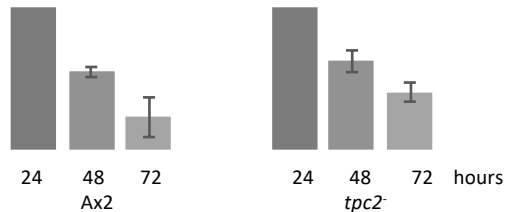

B.

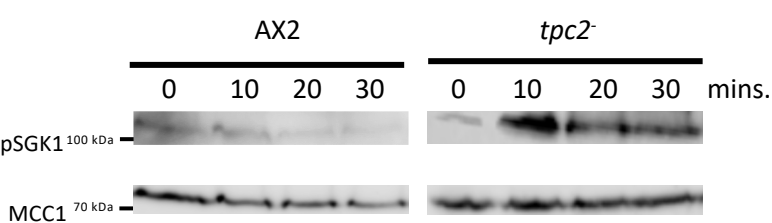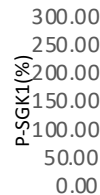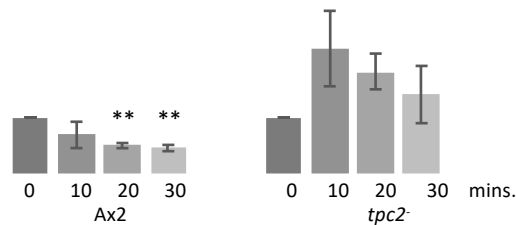

Supplement: Supplementary file 6 — Additional file 6: Figure S6. Western blot analysis of Ax2 and tpc2− cells using anti-phospho SGK1 (Ser 422) as an mTOR substrate. A. Ax2 and tpc2− cells were seeded at a density of 0.7 x 10^7^ cells/ml in HL5 and grown in shaking suspension at 220C. Cells were harvested at the times shown. Whole cell lysates were immunoblotted using antibodies against phospho-SGK1 Ser422. MCCC1 was used as a loading control . One experiment representative of three is shown. The level of phosphorylated protein was quantified with Image Studio Lite (LI-COR) and the average of three independent experiments is shown. B. Exponentially growing parental Ax2 and tpc2− cells were harvested and resuspended at a density of 1.4 x 10^7^ cells/ml in HKC buffer and incubated at 220C and 120 rpm shaking for the times shown. Whole cell lysates were immunoblotted using antibodies against phospho-SGK1 Ser422. The level of MCCC1 was used as a loading control. One experiment representative of three is shown. The level of phosphorylated protein was quantified with Image Studio Lite (LI-COR) and the average of three independent experiments is shown. Statistical analysis for values at 10, 20 and 30 minutes relative to 0 minutes was conducted using Student t-test. ** P ≤ 0.01. [file 12915_2019_735_MOESM6_ESM.pdf]
